# Case Report: Hypoglycemia Due to Metastatic Insulinoma in Insulin-Dependent Type 2 Diabetes Successfully Treated With 177 Lu-DOTATATE

**DOI:** 10.3389/fendo.2022.906012

**Published:** 2022-05-24

**Authors:** Shejil Kumar, Mariah Melek, Peter Rohl

**Affiliations:** Endocrinology Department, St George Public Hospital, Sydney, NSW, Australia

**Keywords:** hypoglycemia, insulinoma, diabetes, type 2 diabetes, lutate, lutetium, neuroendocrine tumor

## Abstract

We describe a 96-year-old man with insulin-dependent type 2 diabetes mellitus who, despite insulin cessation, presented with recurrent hypoglycemia associated with confirmed inappropriate endogenous hyperinsulinemia. ^68^Ga-DOTATATE-PET/CT scans demonstrated increased uptake in the pancreatic tail with multiple large intensely active liver metastases. Liver biopsy confirmed the diagnosis of well-differentiated metastatic neuroendocrine tumor. He was unsuitable for surgical resection and long-acting somatostatin analog therapy was ineffective. Subsequent management with four cycles of Lutate [177-Lutetium-DOTA^0^-Tyr^3^-octreotate (^177^Lu-DOTATATE)] resulted in resolution of hypoglycemia and ongoing clinical, biochemical, and radiological response 6 years after. This case is unique due to not only the paradoxical entity of insulinoma in insulin-dependent diabetes but also the positive sustained outcome after ^177^Lu-DOTATATE, given that unresectable metastatic insulinoma carries a poor prognosis. We review published cases of metastatic insulinoma in patients with diabetes mellitus as well as the literature to-date investigating efficacy and safety of Lutate therapy in metastatic insulinoma.

## Introduction

Although insulinomas are the most common functioning pancreatic neuroendocrine tumors (panNETs) and most common cause of endogenous hyperinsulinemic hypoglycemia, they are rare, occurring in approximately one to four per million people annually (1, 2). Insulinoma is exceptionally rare in a patient with pre-existing diabetes mellitus but important not to miss as a cause of recurrent hypoglycemia when iatrogenic causes have been excluded. Insulinomas are predominantly non-metastatic (90%–95%), sporadic, solitary, small (<2cm), and intrapancreatic NETs and most commonly occur in the fifth to sixth decades of life with equal sex distribution (3, 4). Surgical resection is the only cure, with both cure rates and 10-year survival rate >90% in patients with non-metastatic insulinoma following resection (5, 6).

Metastatic insulinoma, however, carries a poor prognosis. A large European registry including 81 patients with metastatic insulinoma reported a 5-year survival rate of 55.6% (7). Data from an American registry of patients (n = 121) revealed a much lower 5-year survival rate in unresectable metastatic insulinoma compared to those who underwent surgery (14% vs. 84%, p < 0.001) (8). Management of recurrent hypoglycemia in these patients is extremely challenging given lack of definitive surgical cure and limitations of available medical options including paucity of data in insulinoma specifically, modest efficacy, and treatment-related side effects and toxicity (9). ^177^Lu-DOTATATE (Lutate) has an emerging evidence basis in patients with gastroenteropancreatic (GEP) NETs and shows promise in managing hypoglycemia secondary to metastatic unresectable insulinoma; however, further studies are required (10, 11).

We present a 96-year-old man with insulin-dependent type 2 diabetes mellitus (T2DM) and recurrent refractory life-threatening hypoglycemia secondary to metastatic insulinoma. ^177^Lu-DOTATATE resulted in resolution of hypoglycemia and reduction in metastatic disease burden, with ongoing clinical, biochemical, and radiological response at 6-year follow-up. This case is unique due to not only the paradoxical entity of insulinoma in insulin-dependent diabetes but also the positive sustained well-documented outcome after ^177^Lu-DOTATATE, given that unresectable metastatic insulinoma carries a poor prognosis. We review the few published cases of metastatic insulinoma in patients with diabetes and detail the limited existing data investigating ^177^Lu-DOTATATE therapy in patients with metastatic insulinoma.

## Case Description

A 96-year-old man was referred to our Endocrinology institution by his general practitioner for difficult management of longstanding T2DM. He had been experiencing recurrent “funny turns” (six episodes in the preceding 12 months) associated with hypoglycemia culminating in an episode of loss of consciousness necessitating hospitalization. During this period, there was significant reduction in diabetic regimen intensity including insulin cessation; however, weight had remained stable. His hypoglycemic episodes were predominantly fasting and relieved with carbohydrate consumption.

## Diagnosis, Treatment, and Outcomes

Common differentials such as renal/liver failure (eGFR, 55 ml/min/1.73 m^2^), hypocortisolemia, growth hormone deficiency, and malabsorption were excluded. He subsequently underwent a 75-g oral glucose tolerance test (OGTT), which indicated abnormal insulin physiology. The OGTT results (**Table 1A**) revealed inappropriate fasting hyperinsulinemia (23 µIU/ml) in the setting of fasting hypoglycemia (2.6 mmol/L). This was followed by significant hyperglycemia 2 hours after glucose load (15.8 mmol/L) and insufficient insulin response (61 µIU/ml). After 5 hours, he again demonstrated inappropriate hyperinsulinemia (24 µIU/ml) with hypoglycemia (2.5 mmol/L). Thus, the 75-g OGTT indicated a dysregulated relationship between glucose and insulin concentrations, with inappropriate hyperinsulinemia in the setting of hypoglycemia at fasting and 5 hour after glucose load and with significant hyperglycemia and insufficient insulin response at 2 hours after glucose load. During prolonged inpatient fast, several episodes of hypoglycemia occurred during which inappropriate endogenous hyperinsulinemia was confirmed with elevated insulin and C-peptide concentrations during hypoglycemia (**Table 1B**). Sulfonylurea use and insulin antibodies were excluded, raising suspicion for an insulinoma.

**Table 1A:** Paired serum glucose and insulin concentrations during 5-hour 75-g oral glucose tolerance test (OGTT).

	Glucose (mmol/L)	Insulin (mU/L)
**Fasting**	2.6	23
**0.5 hours**	6.3	30
**1 hour**	11.3	47
**1.5 hours**	14.1	57
**2 hours**	15.8	59
**2.5 hours**	14.4	61
**3 hours**	11.2	49
**3.5 hours**	7.4	40
**4 hours**	5.0	33
**4.5 hours**	3.6	29
**5 hours**	2.6	24

Normal range for glucose fasting, 3.6–6.0 mmol/L; normal range for insulin fasting, <10 mU/L; normal range for glucose 2 hours after glucose load, 3.6–7.7 mmol/L.

**Table 1B:** Paired glucose, C-peptide, insulin, and proinsulin concentrations during inpatient fasting episodes of hypoglycemia.

Study	Result	Sample 1	Sample 2	Normal Range (Units)
**Glucose**	Low	2.4	1.0	3.0–5.5 (mmol/L)
**C-peptide**	High	11.02	8.9	0.4–4.5 (ng/mL)
**Insulin**	High	34.2	29.1	2.6–24.9 (mU/L)
**Proinsulin**	High	>99.9	>99.9	<13.3 (nmol/L)

**Figure 1:**
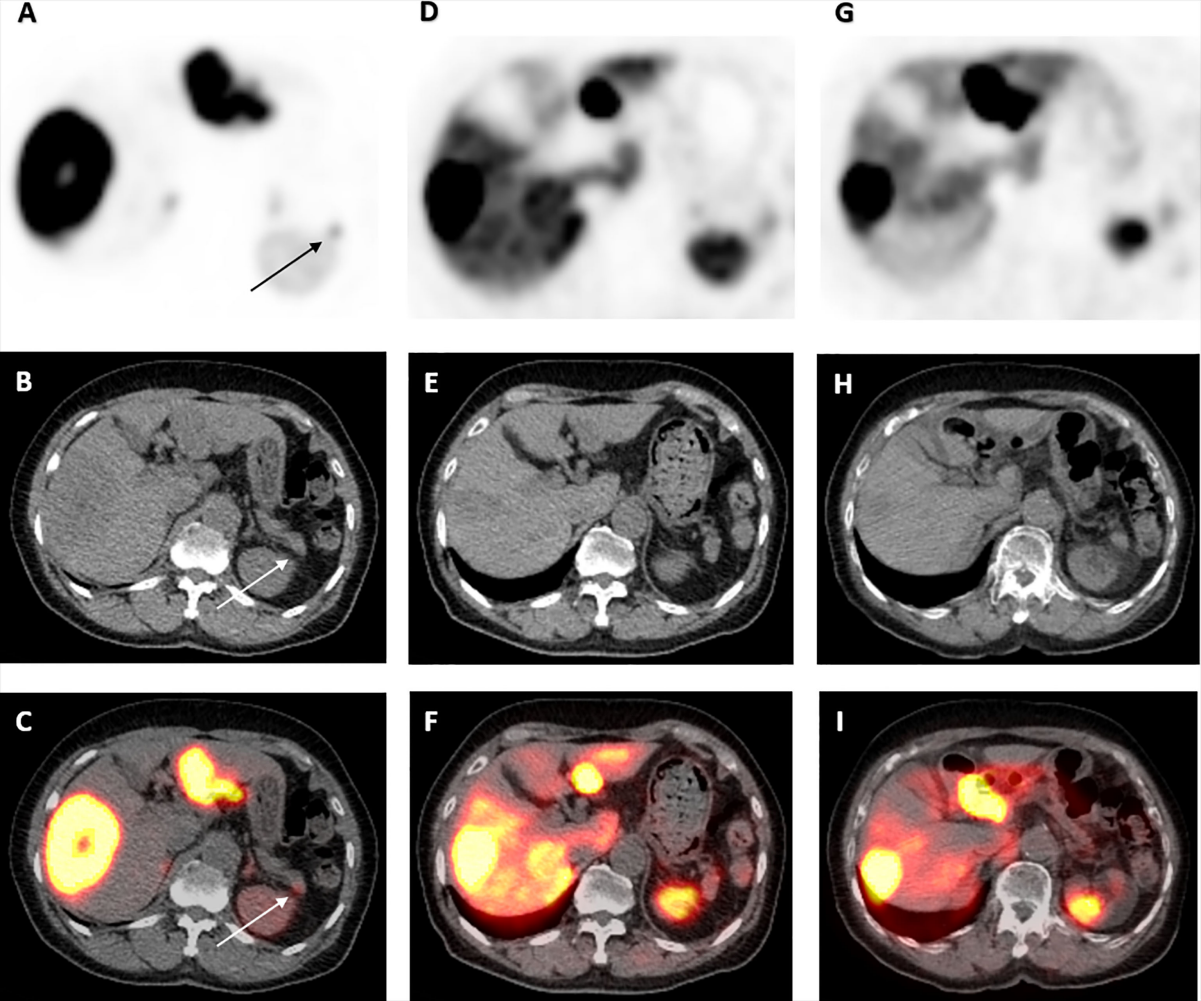
68Ga-DOTATATE PET/CT scan images of pancreatic tail lesion pre- and post-177 Lu-DOTATATE. Axial view images of ^68^Ga-DOTATATE PET/CT scan demonstrating interval reduction in avidity in moderately intense focus of activity at the tip of the pancreatic tail (arrow) on PET, low-dose CT and PET/CT fusion (top to bottom) from baseline, to 1 and 4 years after ^177^Lu-DOTATATE therapy (left to right). Physiological uptake in the spleen and remainder of the liver is also visualized.

**Figure 2:**
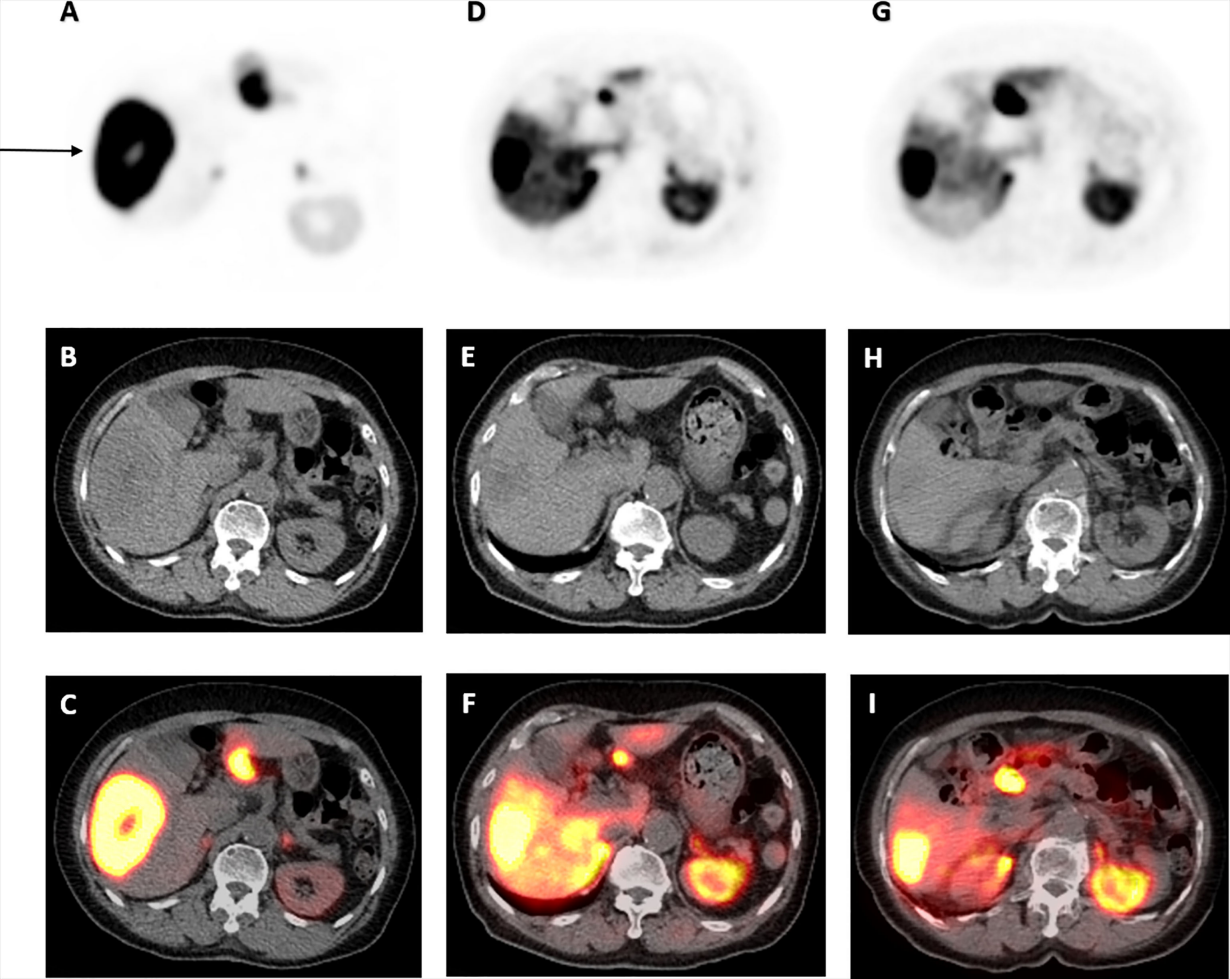
68Ga-DOTATATE PET/CT scan images of dominant right liver lobe lesion pre- and post-177 Lu-DOTATATE. Axial view images of ^68^Ga-DOTATATE PET/CT scan demonstrating interval reduction in size in dominant right liver lobe lesion (arrow) on PET, low-dose CT and PET/CT fusion (top to bottom) from baseline, to 1 and 4 years after ^177^Lu-DOTATATE therapy (left to right). Physiological uptake in the spleen and remainder of the liver is also visualized.

**Figure 3:**
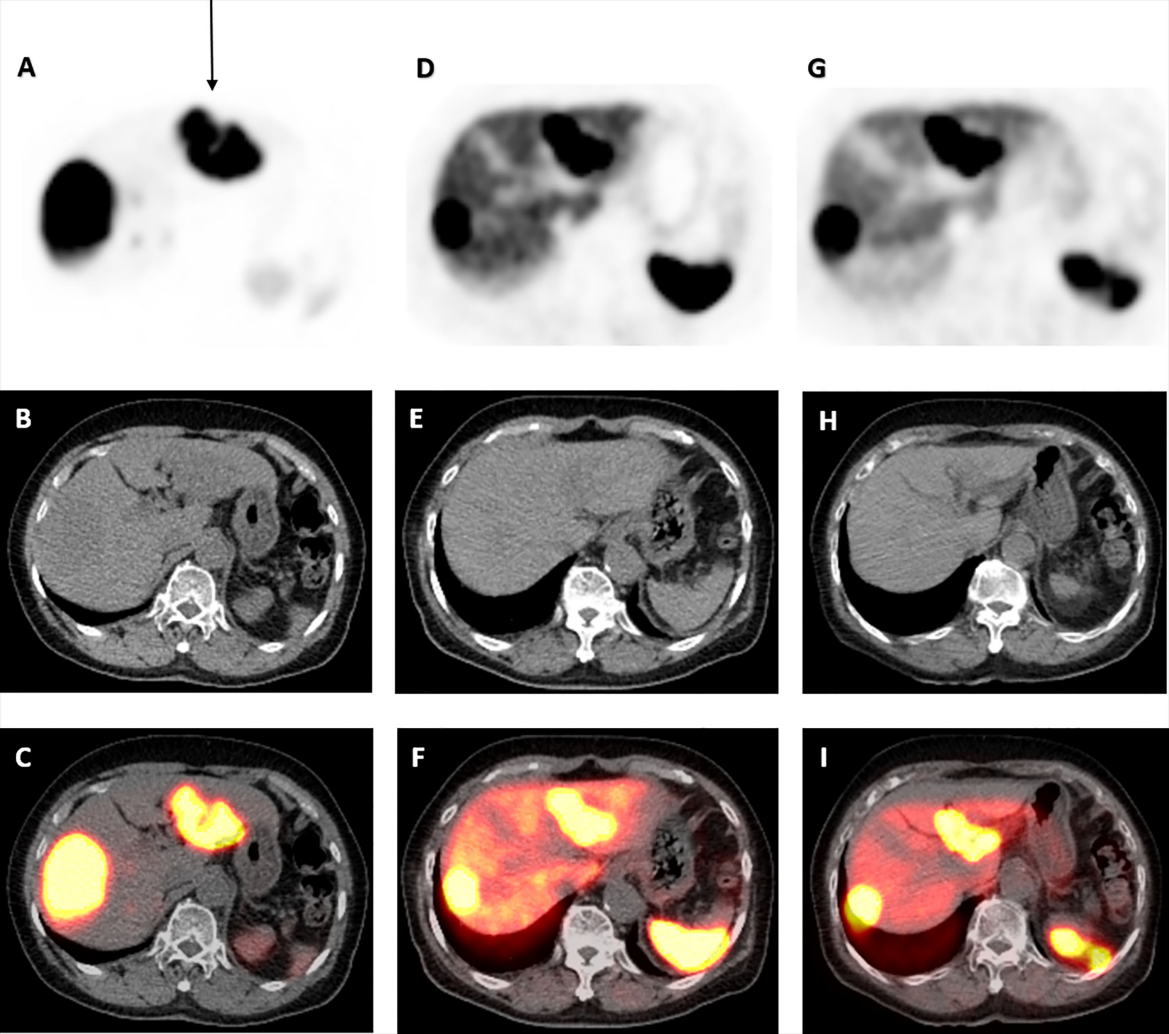
68Ga-DOTATATE PET/CT scan images of dominant left liver lobe lesion pre- and post-177 Lu-DOTATATE. Axial view images of ^68^Ga-DOTATATE PET/CT scan demonstrating interval reduction in size in dominant left liver lobe lesion (arrow) on PET, low-dose CT, and PET/CT fusion (top to bottom) from baseline, to 1 and 4 years after ^177^Lu-DOTATATE therapy (left to right). Physiological uptake in the spleen and remainder of the liver is also visualized.

Computed tomography (CT) scan of the abdomen could not identify focal or diffuse pancreatic enlargement although detected three arterially enhancing liver lesions, measuring 8.0, 6.0, and 2.0 cm. ^68^Gallium-DOTATATE–positron emission tomography (PET)/CT scan, however, showed a small focus of moderately intense tracer accumulation in the pancreatic tail (**Figure 1**) and extensive active metastatic disease in both lobes of the liver (**Figures 2**, **3**). Serum tumor markers α-fetoprotein (AFP), carcinoembryonic antigen (CEA), and cancer antigen (CA)-19.9 were negative. Subsequent liver biopsy revealed a well-differentiated, metastatic NET. Tumor cells were characterized by eccentric round-oval nuclei with mild nuclear pleomorphism and moderate eosinophilic granular cytoplasm. One mitosis per 10 hpf was noted. Tumor cells exhibited strongly positive staining for neuroendocrine markers chromogranin A, synaptophysin, and cluster differentiation (CD)-56, whereas staining was negative for insulin, TTF1, and CDX2. The Ki67 proliferative index was estimated at 2%–3%.

Given advanced age and extensive liver metastases, the patient was deemed unsuitable for surgical resection. He failed initial medical treatment consisting of four weekly intramuscular injections of long-acting Octreotide (30 mg) with another hypoglycemic syncopal event requiring hospital admission. He was then trialed on four cycles of intravenous ^177^Lu-DOTATATE with cumulative dose of 32 GBq over 5 months. He did not experience any dose-limiting side effects such as acute kidney injury or cytopaenia. Hypoglycemic episodes abated, and after a few months, he developed symptomatic hyperglycemia up to 24 mmol/L, which was insufficiently controlled with re-commencement of oral hypoglycemic therapy.

Paradoxically, this elderly man with endogenous hyperinsulinemia from metastatic insulinoma, having had his metastatic disease controlled on ^177^Lu-DOTATATE, began to require exogenous insulin administration for glycemic control.

Repeat ^68^Ga-DOTATATE-PET/CT scans 1 and 4 years after ^177^Lu-DOTATATE completion demonstrated significant durable response with reduction in uptake in the pancreatic tail (**Figure 1**) and throughout the liver (**Figures 2**, **3**). Serial chromogranin A levels also declined from 3,992 to 119 ng/ml (normal range ≤104 ng/ml). He is currently well 6 years after completion of ^177^Lu-DOTATATE for his insulinoma at the age of 96 years with sustained clinical, biochemical, and radiological response.

## Timeline

**Table:** 

April, 2015	Onset of “funny turns”
October, 2015	Endocrinology institution referral
November, 2015	Histopathological diagnosis of metastatic insulinoma
December, 2015	Octreotide commencement
January, 2016	Last serious hypoglycaemic episode
January, 2016	First cycle of ^177^Lu-DOTATATE (8,009 MBq) Octreotide cessation
February, 2016	Second cycle of ^177^Lu-DOTATATE (8,010 MBq)
March, 2016	Resolution of hypoglycemia
April, 2016	Third cycle of ^177^Lu-DOTATATE (7,928 MBq)
June, 2016	Fourth cycle of ^177^Lu-DOTATATE (7,976 MBq)
July, 2016	Onset of symptomatic hyperglycemia
August, 2016	Oral hypoglycemic agent re-commencement
June, 2017	Exogenous insulin re-commencement
January, 2021	Last ^68^Ga-DOTATATE-PET/CT scan
February, 2022	Last clinical assessment

## Discussion

Insulinomas are considered functional panNETs as they result in endogenous hyperinsulinemia, producing the clinical syndrome of recurrent hypoglycemia (most commonly fasting) (1). Whipple’s triad, which is well-documented, serves as the trigger for further investigation (12, 13). Biochemical diagnosis is confirmed with evidence of inappropriate endogenous hyperinsulinemia (elevated/non-suppressed insulin and C-peptide concentrations) during either spontaneous or provoked hypoglycemia (during prolonged inpatient 72-hour fast), after excluding sulfonylurea use (12, 13). Given that surgical resection is the only cure, the next step after confirmed biochemical diagnosis is meticulous localization of the culprit tumor. Localization begins with structural imaging including ultrasound, CT, or magnetic resonance imaging (MRI), followed by functional imaging such as ^68^Ga-DOTATATE-PET/CT scans, which facilitate detection of occult metastases (12, 13). Endoscopic ultrasound (EUS) in combination with fine needle aspiration is the most accurate diagnostic tool for insulinoma with sensitivity and specificity up to 95% and allows histopathological examination of the primary pancreatic tumor and adjacent lymph nodes. EUS, however, is not as sensitive in detecting pancreatic tail tumors (3, 4). Positive tumor cell staining for insulin is supportive but not mandatory for insulinoma diagnosis as up to 20% of patients with pre-operative hyperinsulinemic hypoglycemia and resolution post-tumor resection have negative insulin staining (14). In a retrospective analysis of 80 patients with insulinoma, malignant insulinoma was less likely to stain positive for insulin compared to benign insulinoma (3/7 vs. 66/73, p = 0.015) (15). Hypotheses for lack of insulin staining include defects in insulin storage capacity and sampling errors.

The OGTT is not indicated in the diagnostic algorithm for suspected cases of insulinoma (12), partly due to the occurrence of hypoglycemia during OGTT in some healthy individuals (16). Regardless, the results of a 5-hour OGTT provided the first indication of abnormal insulin physiology in our patient. The OGTT demonstrated inappropriately high fasting insulin during hypoglycemia and insufficient insulin response to hyperglycemia post-glucose ingestion, suggesting that insulin release was occurring largely independent of changes in serum glucose. Under normal physiological conditions, glucose metabolism is intimately coupled with β-cell insulin secretion such that blood glucose concentrations are maintained between 3.5 and 5.5 mmol/L (17), with a sigmoidal pattern of insulin release to higher glucose concentrations in isolated normal human islet cells (18). However, in the presence of an insulinoma, this glucose-insulin coupling becomes dysregulated. Small-scale *in vitro* studies have shown that cultured human insulinoma cells can have near maximal glucose responsiveness at blood glucose levels of 1.0–3.0 mmol/L (19) with no significant increase in insulin release with rise in glucose concentration from 2.8 to 8.3 mmol/L in one study (20) and plateau at 10.0–15.0 mmol/L in two other studies (19, 21). The autonomous insulin secretion despite low glucose concentrations and the blunted insulin response to higher glucose concentrations can give rise to fasting hypoglycemia and postprandial hyperglycemia, respectively. This abnormal insulin secretion pattern was not only evident in our patient but also the most common pattern in a retrospective analysis published in 2008 of 64 patients who underwent 100-g OGTT prior to insulinoma resection (22).

Insulinoma is an exceptionally rare occurrence in patients with diabetes. Among 313 confirmed insulinoma cases at the Mayo Clinic between 1927 and 1992, there was only one patient with pre-existing diabetes (23), whereas a cohort from Japan of 443 cases of insulinoma included one diabetic patient (24). A single institution in Taiwan reported one patient with diabetes out of 23 insulinoma cases seen between 1984 and 2006 (25). Potential explanations for the low reported incidence of diabetes in insulinoma cases include a) lack of reporting in the literature, b) missed or delayed diagnosis due to difficulty differentiating iatrogenic from insulinoma-induced hypoglycemia, c) insulin resistance or pre-existing hypoglycemia unawareness masking the clinical syndrome, or d) decreased β-cell number and thus decreased potential cellular regeneration for tumor formation. Extensive literature review yielded 13 cases of metastatic insulinoma in patients with pre-existing diabetes in the past 50 years (**Supplementary Table 1**) (26–37). The majority had pre-existing T2DM (10/13) with mean age 59 years at diagnosis and equal sex distribution. The most common primary site was the pancreatic tail, and majority (7/8) pancreatic lesions were >4 cm in diameter, compared to mean lesion size of 3 cm in the largest published series of malignant insulinoma cases (8), potentially reflecting delayed diagnosis due to the initial requirement of excluding iatrogenic hypoglycemia. The most common metastatic sites were regional lymph nodes (8/13) and liver (11/13). Diazoxide (9/13) and somatostatin analogs (8/13) were the most common medical therapies utilized, whereas ^177^Lu-DOTATATE has not been reported in a diabetic patient with metastatic insulinoma. Regarding outcomes, six patients died during follow-up, including two patients within 2 weeks from Diazoxide-related toxicity and otherwise due to treatment failure and progressive disease. Six patients had good outcome without recurrence of hypoglycemia; however, this observation is limited by short follow-up length of <1 year in the majority. Thus, our patient shares similarities with previously reported cases of metastatic insulinoma in pre-existing diabetes however is unique given his advanced age, use of ^177^Lu-DOTATATE, and positive sustained outcome at extended 6-year follow-up.

Given that our patient’s advanced age and extensive unresectable liver metastases, curative surgical resection was unsuitable. This led to the difficult medical management issue of recurrent hypoglycemia from metastatic insulinoma. Various review articles have summarized the available medical therapeutic options for managing recurrent insulinoma-induced hypoglycemia (2, 3, 9, 38). Briefly, these strategies are limited by lack of data particularly in patients with insulinoma, modest efficacy, and treatment-related intolerance and toxicity. Diazoxide is a nondiuretic benzothiazide analog, which opens the ATP-sensitive potassium channel on the pancreatic β-cell membrane, hence facilitating potassium cellular efflux and diminishing membrane depolarization and voltage-gated calcium-dependent exocytosis of insulin-containing vesicles (9). Diazoxide has approximately 50% efficacy in abating hypoglycemia in insulinoma but is not useful in controlling metastatic disease (hence not used in our patient) and is often limited by significant toxicity, e.g., fluid retention, renal/liver failure (3). Somatostatin analogs such as long-acting Octreotide and Lanreotide inhibit insulin release from pancreatic β-cells and have up to 50% efficacy in controlling hypoglycemia with modest tumor regression effect. Somatostatin analog use is often limited by gastrointestinal side effects and tachyphylaxis and in some cases worsens hypoglycemia due to concurrent inhibition of counter-regulatory glucagon release (9). Other less investigated but approved options in unresectable GEP NETs include chemotherapy (5-FU, Doxorubicin, Streptozotocin, Temozolomide, and Capecitabine), mTOR inhibitors such as Everolimus and multiple tyrosine kinase inhibitor Sunitinib, with data particularly scarce in metastatic insulinoma (9, 38).

We successfully trialed ^177^Lu-DOTATATE in our patient, a form of peptide receptor radionuclide therapy (PRRT). Lutate (177-Lutetium-DOTA^0^-Tyr^3^-octreotate) is a radiolabeled somatostatin analog compound consisting of the somatostatin analog Tyr^3^-octreotate, linked with a radioactive isotope, 177-Lutetium, with DOTA^0^ acting as the linking agent. Intravenous infusion of ^177^Lu-DOTATATE allows delivery of targeted cytotoxic ionizing radiation therapy specifically to neuroendocrine tumor cells, taking advantage of their somatostatin receptor (SST) overexpression (particularly SST2) and the low physiological SST expression in normal tissue. Compared to earlier radionuclides, 177-Lutetium emits diagnostic ƴ-radiation, allowing better dosimetry, emits therapeutic β-radiation that has a shorter tissue penetration range (2mm), limiting exposure to neighboring normal tissue, and has nine-fold higher affinity for SST2, and lower hematological/renal toxicity (10, 39).

The Rotterdam group in Netherlands has reported results of ^177^Lu-DOTATATE in several patients with NETs in the past 20 years. Kwekkeboom et al. investigated 131 patients with metastatic GEP NETs (including two with insulinoma) with median follow-up 16 months (40). Results were favorable with 47% having an objective response, 35% stable disease, and 18% progressive disease, and ^177^Lu-DOTATATE was considered safe with <2% experiencing serious hematological toxicity and two cases of serious liver/hepatic toxicity. Brabander et al. more recently assessed ^177^Lu-DOTATATE efficacy in 443 patients with metastatic bronchial and GEP NETs (including 21 patients with functional panNETs) with 78-month median follow-up (41). Progression-free survival was 29 months, time to progression of 36 months, and overall survival of 63 months, with objective response (complete or partial) in 39% and stable disease reached in 43% of patients. Clinically significant hematological toxicity occurred in 10%, including acute leukemia (AL) in 0.7% and myelodysplastic syndrome (MDS) in 1.5% of patients with no treatment-related long-term renal or liver failure observed.

However, given the low prevalence of metastatic insulinoma, data exploring efficacy of ^177^Lu-DOTATATE in these patients specifically are limited to small case series and single case reports. Our literature review revealed 33 published cases of metastatic insulinoma trialed on ^177^Lu-DOTATATE therapy, with 32/33 having liver metastases (11, 42–49).

The largest such case series was conducted by Zandee et al. who investigated ^177^Lu-DOTATATE safety and efficacy in 34 patients with metastatic functioning panNETs including 14 with insulinoma (11). Eight patients had pre-treatment with somatostatin analogs, five had surgery, and two had chemotherapy. Objective response, stable disease, and progressive disease occurred in 50%, 21.4%, and 28.6% of patients, respectively, with approximately 30 months mean progression-free survival and 67% experiencing reduction in hypoglycemia frequency.

In a cohort of 310 patients with GEP NETs managed with ^177^Lu-DOTATATE between 2000 and 2006, Kwekkeboom et al. included five patients with metastatic insulinoma with partial response in three patients, stable disease in one patient, and progressive disease in the other (42).

Ong et al. described two men with inoperable metastatic insulinoma with severe hypoglycemia, who failed Diazoxide and somatostatin analog therapy (43). Both patients experienced control of hypoglycemia and reduction in size of liver metastases with ^177^Lu-DOTATATE; however, one was co-treated with Everolimus and both with chemotherapy. One patient was hypoglycaemia-free at 10 months and the other had disease progression at 24 months.

Van Schaik et al. treated four patients with metastatic insulinoma and severe uncontrollable hypoglycemia failing conventional therapy including Octreotide (44). ^177^Lu-DOTATATE achieved stable disease and euglycemia for mean 22 months (one patient still in remission at 20 months).

Magalhães et al. utilized ^177^Lu-DOTATATE in four patients with unresectable metastatic insulinoma and refractory hypoglycemia all pre-treated with Diazoxide and Octreotide (45). Two patients had disease progression (mean 14 months) and mortality (mean 20 months), whereas two patients remained asymptomatic at mean follow-up 20 months.

Four single case reports have also outlined positive effects of ^177^Lu-DOTATATE in metastatic insulinoma with refractory hypoglycemia, such as resolution of hypoglycemia and reduction in metastatic burden with all patients having ongoing disease control and radiological stability at mean follow-up interval of 15 months (46–49).

Hence, ^177^Lu-DOTATATE is a potential effective and safe option in patients with unresectable metastatic insulinoma with perceived benefits including resolution of hypoglycemia and reduction in radiological metastatic burden. However, given likely positive publication bias and scarce data, further studies (ideally randomized and controlled) exploring^177^Lu-DOTATATE efficacy in this subset of patients is certainly warranted.

Insulinoma, although a rare cause of hypoglycaemia in a patient with diabetes mellitus, is important not to miss and should especially be considered in patients on minimal glucose-lowering therapy or in whom hypoglycemia continues despite insulin cessation. Surgical management is the only cure and is preferred in suitable patients, whereas medical management in cases of unresectable insulinoma with recurrent hypoglycemia is extremely challenging due to poor prognosis and limitations of available treatment options such as diazoxide, somatostatin analogs, and chemotherapy. ^177^Lu-DOTATATE, a form of PRRT, has an emerging evidence basis in patients with GEP NETs and shows promise as a potential effective and well-tolerated option in patients with recurrent hypoglycemia secondary to unresectable metastatic insulinoma; however, further studies are needed. Our patient had a successful and sustained response to ^177^Lu-DOTATATE to the extent that he is now requiring exogenous insulin administration for his previously masked poor diabetic control. Despite not undergoing surgical management, ^177^Lu-DOTATATE has provided him an exceptional outcome in terms of survival and quality of life considering his advanced age and extent of liver metastases.

## Patient Perspective

The patient declined to provide their perspective on the case report, however, provided signed written informed consent for this report to be published.

## Data Availability Statement

The original contributions presented in the study are included in the article/**Supplementary Material**. Further inquiries can be directed to the corresponding author.

## Ethics Statement

Ethical review and approval was not required for the study on human participants in accordance with the local legislation and institutional requirements. The patients/participants provided their written informed consent to participate in this study. Written informed consent was obtained from the individual(s) for the publication of any potentially identifiable images or data included in this article.

## Author Contributions

SK conceived the case report and drafted and critically reviewed the manuscript. MM drafted the manuscript. PR managed the patient and critically reviewed the manuscript. All authors contributed to the article and approved the submitted version.

## Funding and Acknowledgments

The authors have no funding or acknowledgments to disclose.

## Conflict of Interest

The authors declare that the research was conducted in the absence of any commercial or financial relationships that could be construed as a potential conflict of interest.

## Publisher’s Note

All claims expressed in this article are solely those of the authors and do not necessarily represent those of their affiliated organizations, or those of the publisher, the editors and the reviewers. Any product that may be evaluated in this article, or claim that may be made by its manufacturer, is not guaranteed or endorsed by the publisher.
