# Supplementary material for: Case Report: Hypoglycemia Due to Metastatic Insulinoma in Insulin-Dependent Type 2 Diabetes Successfully Treated With 177 Lu-DOTATATE
Source: Front Endocrinol (Lausanne). 2022 May 24;13:906012. doi: 10.3389/fendo.2022.906012 (PMC9171402; doi:10.3389/fendo.2022.906012)
Supplement: Supplementary file 1 [file Table_1.docx]

**SUPPLEMENTARY TABLE 1 – Summary of reported patients to-date with metastatic insulinoma and pre-existing diabetes mellitus**

| **Authors** | **Year** | **Age, Sex** | **Site** | **Size** | **Metastases** | **Localization** | **Treatment** | **Outcome** |
| --- | --- | --- | --- | --- | --- | --- | --- | --- |
| Kumar et al. | 2022 | 90, M T2DM | Tail | NR | Liver | Abdomen CT SSTR scintigraphy | Octreotide Lutate | Resolution of hypoglycemia with Lutate Resumed insulin treatment No recurrence and stable liver metastases at 6 years post-Lutate treatment |
| Ciacciarelli et al. | 2020 | 66, M T2DM | Tail | 4.3 x 2.2 cm | Regional lymph nodes Liver | Whole-body CT | Diazoxide | Death <2 weeks after diagnosis due to diazoxide renal toxicity |
| Gjelberg et al. | 2017 | 43, F T1DM | Tail | 11.0 x 7.0 cm | Retroperitoneal lymph nodes  Liver Peritoneum | Abdomen US Abdomen CT SSTR scintigraphy | Surgical resection Streptozotocin/5-FU  Octreotide  Interferon  Chemoembolization  Temozolomide  Capecitabine | Recurrent disease progression and various intolerances of several lines of therapy  Death 11 years after surgical resection with peritoneal carcinomatosis |
| Lablanche et al. | 2015 | 31, M T1DM | Head | 6.3 x 5.6 cm | Regional lymph nodes | Abdomen CT SSTR scintigraphy | Surgical resection | Resumed insulin treatment No recurrence of hypoglycemia or tumor relapse for 4-year follow-up |
| Ademoglu et al. | 2012 | 45, F T2DM | Head | 0.9 x 1.0 cm 0.8 x 0.7 cm 0.7 x 0.7 cm | Regional lymph nodes | EUS | Surgical resection | Resolution of hypoglycemia  Commencement of insulin and oral hypoglycemic agents for T2DM No recurrence at 3-months |
| Abbasakoor et al. | 2011 | 67, F T2DM | Body Tail | 5.0 x 6.0 cm 2.6 x 2.0 cm | Regional lymph nodes Liver | Abdomen CT EUS  SSTR scintigraphy | Diazoxide Octreotide  Surgical resection | Resolution of hypoglycemia  Recurrence-free at 9-months post-operatively |
| Ferrer-Garcia et al. | 2011 | 78, M T2DM | Head | 4.7 x 3.0 cm | Retroperitoneal / mesenteric lymph nodes Mesenteric vein Liver | EUS Abdomen CT Abdomen MRI SSTR scintigraphy | Chemoembolization Octreotide  Diazoxide  Dexamethasone Everolimus | Recurrent hypoglycemia until control after 6 months of Everolimus Resumed insulin treatment Stable liver metastases |
| Grycewicz et al. | 2010 | 60, F T2DM | NF | NF | Para-aortic / mesenteric lymph nodes  Liver | Abdomen US Abdomen CT SSTR scintigraphy | Octreotide Y-90 DOTATATE Diazoxide | Resolution of hypoglycemia with Diazoxide Commencement of insulin treatment No recurrence at 6-months |
| Campos-Olive et al. | 2010 | 76, M T2DM | Head | 4.7 x 3.0 cm | Retroperitoneal / mesenteric lymph nodes Liver Superior mesenteric vein | Abdomen CT Abdomen MRI EUS SSTR scintigraphy | Chemoembolization Octreotide  Diazoxide | Improvement of hypoglycemia with Diazoxide  Persistent mild hypoglycemia 12-months after diagnosis |
| Schmitt et al. | 2008 | 79, F T2DM | Body Tail | NR | Liver | Abdomen US Abdomen CT | Diazoxide Lanreotide | Death 2 weeks after diagnosis due to diazoxide related renal and liver toxicity and hyponatremia |
| Siraj et al. | 2006 | 74, F T2DM | Tail | 7.3 x 5.7 cm | Liver | Abdomen CT  Abdomen MRI | Surgical resection Diazoxide Octreotide | Death 3 years after diagnosis with progressive metastatic disease and recurrent hypoglycemia |
| Svartberg et al. | 1996 | 33, F T1DM | Tail | NR | Liver Right ovary | Exploratory laparotomy | Debulking surgery Diazoxide Interferon Streptozotocin Doxorubicin Octreotide | Recurrent hypoglycemia failing multiple lines of therapy  Death 2 years after diagnosis |
| Atkinson et al. | 1978 | 43, M T2DM | NR | NR | Liver | Laparotomy | Streptozotocin | Resumed insulin Recurrence of hypoglycemia at 12 months Death 18 months after initial treatment |
| Taylor et al. | 1970 | 70, M T2DM | Tail | NR | Liver | Laparotomy | Surgical resection Diazoxide Prednisone Streptozotocin | Resolution of hypoglycemia with Streptozotocin Resumed insulin treatment  No recurrence at 8-months post Streptozotocin |

NR = not reported; NF = not found; M = male; F = female; T2DM = type 2 diabetes mellitus; T1DM = type 1 diabetes mellitus; CT = computed tomography; US = ultrasound; MRI = magnetic resonance imaging; SSTR = somatostatin receptor; 5-FU = 5-fluorouracil.

This table summarises the 14 cases published to-date (including our case) of metastatic insulinoma in pre-existing diabetes in reverse chronological order, including authors, year of publication, patient demographics and type of diabetes, location and size of pancreatic primary, distribution of metastases, localization strategies, treatments and outcomes.
